# Conotoxin Prediction: New Features to Increase Prediction Accuracy

**DOI:** 10.3390/toxins15110641

**Published:** 2023-11-03

**Authors:** Lyman K. Monroe, Duc P. Truong, Jacob C. Miner, Samantha H. Adikari, Zachary J. Sasiene, Paul W. Fenimore, Boian Alexandrov, Robert F. Williams, Hau B. Nguyen

**Affiliations:** 1 Bioscience Division, MS M888, Los Alamos National Laboratory, Los Alamos, NM 87545, USA; 2 Theoretical Division, MS M888, Los Alamos National Laboratory, Los Alamos, NM 87545, USA

**Keywords:** conotoxins, machine learning, collisional cross section, post-translational modifications, prediction, ion mobility–mass spectrometry

## Abstract

Conotoxins are toxic, disulfide-bond-rich peptides from cone snail venom that target a wide range of receptors and ion channels with multiple pathophysiological effects. Conotoxins have extraordinary potential for medical therapeutics that include cancer, microbial infections, epilepsy, autoimmune diseases, neurological conditions, and cardiovascular disorders. Despite the potential for these compounds in novel therapeutic treatment development, the process of identifying and characterizing the toxicities of conotoxins is difficult, costly, and time-consuming. This challenge requires a series of diverse, complex, and labor-intensive biological, toxicological, and analytical techniques for effective characterization. While recent attempts, using machine learning based solely on primary amino acid sequences to predict biological toxins (e.g., conotoxins and animal venoms), have improved toxin identification, these methods are limited due to peptide conformational flexibility and the high frequency of cysteines present in toxin sequences. This results in an enumerable set of disulfide-bridged foldamers with different conformations of the same primary amino acid sequence that affect function and toxicity levels. Consequently, a given peptide may be toxic when its cysteine residues form a particular disulfide-bond pattern, while alternative bonding patterns (isoforms) or its reduced form (free cysteines with no disulfide bridges) may have little or no toxicological effects. Similarly, the same disulfide-bond pattern may be possible for other peptide sequences and result in different conformations that all exhibit varying toxicities to the same receptor or to different receptors. We present here new features, when combined with primary sequence features to train machine learning algorithms to predict conotoxins, that significantly increase prediction accuracy.

## 1. Introduction

Conotoxins are peptides found in the venom of carnivorous aquatic mollusks known as cone snails that hunt by paralyzing their prey [1]. This happens because conotoxins interfere with the normal function of various ion channels and signal receptors, ultimately leading to paralysis and suffocation [1]. Despite the risks to human health posed by these kinds of toxins, there are limited effective anti-toxins available. The rational development of novel therapeutics requires topological knowledge of the receptors, binding sites, and interacting residues for a given toxin [2]. There is increasing interest in peptide-based toxins for medical use as treatments for cancers [3,4], microbial infections [5], epilepsy, autoimmune diseases, neurological conditions, and cardiovascular disorders [4,6]. As an example, the drug Ziconotide (Prialt), used for chronic pain relief, is a synthetic version of the ω-conotoxin MVIIA from the cone snail, *Conus magus* [7].

**Figure 1:**
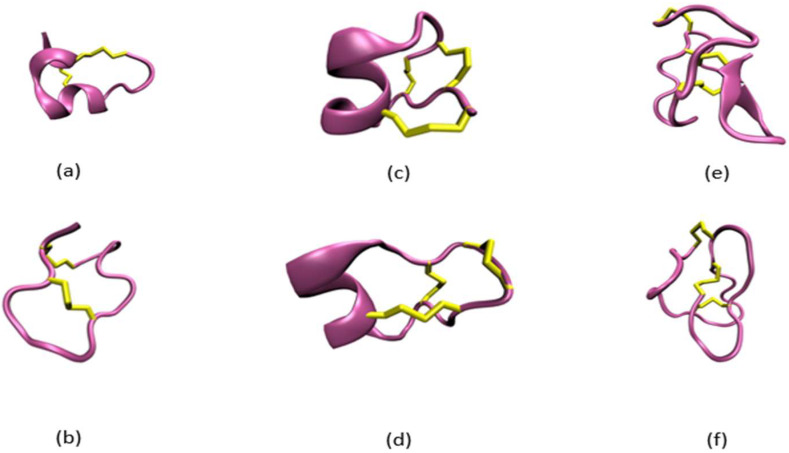
Example of conotoxins adopting different structures while sharing the same primary amino acid sequence (**a**–**d**) or conotoxins sharing the same disulfide bond pattern but different primary amino acid sequences (**e**,**f**). (**a**) Alpha conotoxin AuIB in its native conformation with a disulfide bond pattern of Cys2-Cys8 and Cys3-Cys15 (PDB: 1MXN [18]). (**b**) Alpha conotoxin AuIB in its ribbon (isoform) conformation with a disulfide bond pattern of Cys2-Cys15 and Cys3-Cys8 (PDB: 1MXP [18]). (**c**) Mu conotoxin KIIIA with a disulfide bond pattern of Cys1-Cys9, Cys2-Cys15, and Cys4-Cys16 (PDB: 7SAV [19]). (**d**) Mu conotoxin KIIIA with a disulfide bond pattern of Cys1-Cys16, Cys2-Cys9, and Cys4-Cys15 (PDB: 7SAW [19]). (**e**) Kappa conotoxin PVIIA with a disulfide bond pattern of Cys1-Cys16, Cys8-Cys20, and Cys15-Cys26 (PDB: 1AV3 [20]). (**f**) Omega conotoxin MVIIA with a disulfide bond pattern of Cys1-Cys16, Cys15-Cys25, and Cys8-Cys20 (PDB: 1DW4 [21]).

Despite recognizing the importance of these dual-use compounds as both dangerous and potentially therapeutic, our ability to identify, characterize, and determine the toxicities of conotoxins is difficult, costly, and time-consuming. Overcoming this challenge requires a series of diverse, complex, and labor-intensive biological, toxicological, and analytical techniques for effective characterization [8]. Furthermore, with the thousands of new peptide sequences that are being obtained by transcriptomics and proteomics, traditional toxicological measurements are too slow. In many cases, the experimental determination of an individual toxin’s function has become unfeasible and/or impossible because of the time-consuming nature and high cost of the experiments. Recent attempts using deep learning or machine learning (ML) (i.e., TOXIFY [9], ToxClassifier [10], ClanTox [11], ToxinPred [12], PredCSF [13]) to predict the toxicity of peptides (e.g., conotoxins and animal venoms, etc.) based on primary amino acid sequences have improved the toxicity identification process. However, these methods are limited due to the inherent conformational heterogeneity exhibited by peptides, which comes from two primary sources: [1] the innate flexibility of peptides or peptide backbone and [2] the proportionally high numbers of cysteines. High cysteine counts allow peptides to adopt multiple conformational permutations that are stabilized by the disulfide bonds formed between the cysteine pairs. Peptide sequences locked into different conformations lead to shifts in physiological behavior by providing different interaction surfaces (i.e., topology). A given peptide may be toxic when its cysteine residues form a particular disulfide-bond pattern, resulting in a specific conformation. Alternative disulfide-bonding patterns, such as when the conotoxin peptide is an isoform (containing alternative cysteine–cysteine disulfide bridges) or is in its reduced form (absence of any disulfide bridges), may yield little or no toxicological effects for the peptide or may be highly toxic (Figure 1). For example, the conotoxin AuIB has an IC_50_ value of 1.2 nM in its native helical form (Figure 1a), but the IC_50_ decreases by a factor of 10 (to 0.1 nM) when AuIB is converted to its ribbon (Figure 1b) isoform [14]. In contrast, the conotoxin GI in its native form shows a 10-fold greater IC_50_ compared to its two ribbon isoforms [15]. Consequently, similar disulfide-bond patterns result in different conformations for different peptide sequences, which all exhibit varying toxicities from altered binding to the same receptor or binding to different receptors. Furthermore, while the majority of conotoxins contain post-translationally modified (PTM) amino acids (e.g., hydroxyproline, pyroglutamic acid, etc.), all current prediction methods and models exclude these unique residues, categorizing them as their unmodified residues. This exclusion results in a decrease in the potential size of a unique dataset and tremendously reduces the effectiveness and accuracy of any predictions [13,16,17].

To augment the predictive capability of ML approaches for conotoxin prediction, we integrated a variety of physiochemical and structural features, including physiochemical surface properties, secondary structure characteristics, and collisional cross sections (CCSs). A CCS is an experimental value obtained from ion mobility–mass spectrometry (IM-MS) experiments or from a computational calculator such as the High-Performance Collision Cross Section (HPCCS) [22] software. The experimental or computational CCS value is a function of the size, shape, charge, and polarizability of a molecule. Here, we determine how these additional features improve conotoxin prediction accuracy and how to include them in building an effective ML-based platform to accurately predict if an unknown toxin molecule is a conotoxin. Such a platform will not only accelerate the identification of novel biochemical threat agents but also benefit the development of biological prophylactics and therapeutics, detection reagents, and medical countermeasures.

## 2. Results

### 2.1. Construction of Datasets

In order to evaluate how new features impact conotoxin prediction accuracy, negative datasets were separated into **easy-negative** and **hard-negative** datasets. The easy-negative dataset contains peptides (from more than 100 species, including humans, yeast, zebrafish, mice, eels, chickens, and cattle) that are confirmed to be non-toxic, while the hard-negative dataset contains toxic peptides from spiders, scorpions, snakes, beetles, frogs, wasps, and ants, as well as conantokins and contryphans from cone snails. Toxic peptides were categorized as part of the hard-negative dataset with the expectation that they may contain similar amino acid compositions (regions) and also share similar binding sites with conotoxins; thus, they would be more difficult to distinguish from the conotoxins.

In general, three datasets: a positive, an easy-negative, and a hard-negative dataset, were constructed for the training and testing of the ML approach (see the methods section). These three datasets were initially collected from the Protein Data Bank (PDB) using the keywords indicated in the methods section. The positive datasets include conotoxins obtained from the Conoserver, PDB, and the Biological Magnetic Resonance Bank websites. These conotoxins are from twelve (12) distinct classes, including the alpha, delta, mu, and omega classes, that target nicotinic acetylcholine receptors (nAChRs), GABA receptors, and potassium (K^+^), calcium (Ca^2+^), and sodium (Na^+^) ion channel receptors. A distribution of these classes is shown in Figure S1. We initially constructed small-sized datasets that include a positive dataset containing 154 conotoxins, an easy-negative dataset containing 180 non-toxic peptides, and a hard-negative dataset containing 178 peptides. To test how consistently the new features affect conotoxin prediction accuracy, we expanded our datasets by adding more entries into these small datasets. The extended datasets include a positive dataset containing a total of 184 conotoxins, an easy-negative dataset containing 317 non-toxic peptides, and a hard-negative dataset containing 560 peptides. All the entries in these datasets are peptides with lengths equal to or less than 80 residues and with known three-dimensional structures. Sequences with more than a 90% sequence identity were removed from the negative datasets. A summary of all the datasets collected and used for the ML experiments is shown in Table 1. A full list of these datasets along with all the features extracted is available in File S1.

### 2.2. Feature Extraction and Selection

**Figure 2:**
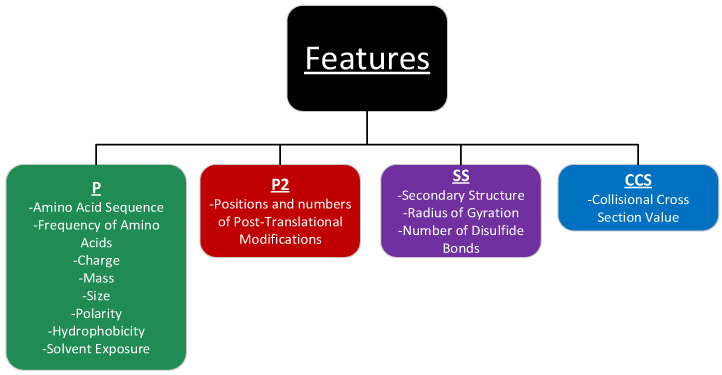
Features were divided into four groups (P, P2, SS, and CCS), and the effect of each feature group was evaluated with regard to conotoxin prediction accuracy.

Thirteen features belonging to three general categories (compositional, physiochemical, and structural) were divided into four groups (P, P2, SS, and CCS) (Figure 2). The compositional features consist of the peptide amino acid sequence, the frequency of amino acid occurrence, both of which have been used for standard biomolecular classifications [23,24,25], and the number of post-translational modifications (PTMs). Since conotoxins show high concentrations of PTMs, this is an important feature that has not yet been considered for improving prediction accuracy. New parameters for some common PTMs are found in Table S1. The physiochemical features include protein charge, mass, size, relative polarity, and hydrophobicity as shown in Table S2. The structural features inform peptide folds and include secondary structure identities, the radius of gyration, disulfide bond counts, and solvent-accessible surface areas (SASA). Because conotoxin function depends on surface topological interactions, we hypothesize that by characterizing the chemical surface of conotoxins, we should see an improvement in classification. Therefore, the SASA of each residue on the peptide was quantified. In addition, in order to test if an unknown conotoxin can be quickly and accurately predicted using an experimental parameter such as the CCS value obtained from an IM-MS experiment, we added the computationally calculated CCS values to the list of features for ML prediction. Additional details on the features are included in Supplemental Tables S2 and S3, and a visual aid for the surface characteristics is shown in Figure S2.

To determine the performance of the feature groups on conotoxin prediction, each feature group was, either individually or in combination with other feature groups, evaluated for efficacy. The features were split into four groups, identified as P, P2, SS, and CCS, as shown in Figure 2. The feature group P contains the counts for 11 sequence-level features (aliphatic, aromatic, polar, hydrophobic, charged, positively charged, negatively charged, tiny, small, large, and total), as well as total charge, mass, dipeptide 0 gap, and dipeptide 1 gap. Dipeptide 0 and dipeptide 1 are the frequencies of co-occurring residues in the sequence as adjacent neighbors or with one residue separating them, respectively. Most of the current ML models use only the P features to train ML algorithms [26]. The feature group SS contains the residue counts for each of the defined secondary structures extracted from the Define Secondary Structure of Proteins DSSP [27,28] program (Table S3), namely disulfide-bond count, the radius of gyration, and the SASA of the residue types, including the PTMs. The feature group P2 contains the counts of PTMs and the frequency of the dipeptide 2 gap, which is the frequency of residues appearing as neighbors with two residues separating them. The feature group CCS contains the CCS values of all the entries calculated by the HPCCS program using the corresponding structure files from the PDB. A list of all feature sets is included in Table S4.

### 2.3. Effect of Features on Prediction Performance

One common challenge with training ML models on biological samples is that the dataset sizes are usually small because the available experimental biological data are very limited. This is especially true for the positive datasets. In contrast, the negative datasets have considerably more entries due to their diversified sequences and the availability of more experimental data.

#### 2.3.1. Improved Predicting Power from New Features across All Datasets

In order to determine how the PTMs, structural features, and CCS features affect classification performance, these features were tested on small and extended datasets (Table 1) using four different ML classifiers: a Penalized Logistic Regression (PLR) [29], a Support Vector Machine (SVM) [30], a Random Forest (RF) [31], and XGBoost [32]. Due to the small positive dataset size, the models were tested using a leave-one-out cross validation in which the models were trained using all but one entry and then tested with the entry that was left out, which is then repeated, leaving a different entry out each time [29]. The best average accuracy (AA) and f1 scores obtained from these four classifiers are shown in Table 2. The addition of the CCS, P2, and SS features was shown to consistently increase the classifiers’ AA performance by between 0.08 and 2.8%, with f1 scores increasing by up to 0.0253 across small and extended datasets, with more impact on the conotoxins vs. easy + hard-negative datasets, indicating that these features are highly beneficial for predicting conotoxins.

#### 2.3.2. Conotoxin Prediction Accuracy

Additional detailed testing was evaluated using the extended datasets to reveal the effect of individual feature sets and different feature set combinations on ML classification performance. Three metrics (overall accuracy (OA), average accuracy (AA), and f1 score (f1)) were used to evaluate the classification performance, as indicated in the methods section. Higher values for these metrics indicate a better performance of the classifier.

Table 3 shows the performance of the PLR and SVM classifiers (Top) and RF and XGBoost classifiers (Bottom) for predicting conotoxins against the easy-negative dataset (non-toxic peptides). The results show that the SS features alone, or in combination with the CCS feature did not increase the prediction accuracy and f1 score compared to P features alone. Similarly, the addition of the CCS feature on top of the P features (P + CCS), the addition of SS features on top of the P2 features (SS + P2), or the addition of the CCS and SS features on top of the P2 features (CCS + SS + P2) did not significantly affect the performance of all four classifiers. However, adding the SS features on top of the P feature set (P + SS) increased all three metrics for the PLR, SVM, and XGBoost classifiers, while almost no change was observed for the RF classifier. In particular, the OA was increased by 1.34%, the AA by 2.52%, and the f1 score by 0.0241 for the PLR classifier; the OA by 1.75%, the AA by 1.69%, and the f1 score by 0.0417 for the SVM classifier; and the OA by 1.08%, the AA by 1.37%, and the f1 score by 0.0241 for the XGBoost classifier. Interestingly, adding the P2 features on top of the P features (P + P2) increased all three metrics for all four classifiers: PLR, SVM, RF, and XGBoost. Specifically, for the PLR classifier, adding the P2 feature (P + P2) increased the OA by 2.15%, the AA by 4.1%, and the f1 score by 0.0397, and these numbers are quite similar for the SVM classifier (the OA increased by 2.15%, the AA increased by 3.1%, and the f1 score increased by 0.0473). For the XGBoost classifier, smaller increases were observed, for which the OA increased by 1.21%, the AA increased by 1.81%, and the f1 score increased by 0.0263, while these numbers are slightly lower for the RF classifier, for which the OA increased by 0.53%, the AA increased by 0.59%, and the f1 score increased by 0.0123.

When all the features were combined (P + SS + CCS + P2), the best performance was obtained across all four classifiers and over all three metrics, with converged numbers of an OA of ~96%, an AA of ~95%, and an f1 score of ~0.92. The combination of the features significantly improved the performance of the PLR and SVM classifiers, with an increase in the OA by 3.36%, the AA by 5.58%, and the f1 score by 0.0668 for the PLR classifier, while the SVM classifier saw an OA increase of 2.55%, an AA increase of 3.1%, and an f1 score increase of 0.0579. These increases are slightly smaller for the XGBoost classifier, with an OA increase of 1.35%, an AA increase of 2.1%, and an f1 score increase of 0.029, while for the RF classifier, only a slight improvement was observed, with an OA increase of 0.27%, an AA increase of 0.39%, and an f1 score increase of 0.0059. This result is consistent with the addition of P2 features on top of the P features (P + P2), where larger improvements were made for the PLR and SVM classifiers and only a slight improvement was obtained for the XGBoost classifier, with the least improvement observed for the RF classifier.

Similar to the predictions of conotoxins against the easy-negative dataset, the addition of the P2 features on top of the P features (P + P2) increases the overall performance across the PLR, SVM, RF, and XGBoost classifiers in predicting conotoxins against the hard-negative dataset (other toxic peptides), as shown in Table 4. When all the features are combined (P + SS + CCS + P2), the performance again improves over all three metrics and across all four classifiers when compared to just using P as the only feature. Both the PLR and SVM classifiers show similar increases of 1.55% for the OA, 2.21% for the AA, and 0.0255 for the f1 score; and 1.51% for the OA, 2.31% for the AA, and 0.0262 for the f1 score, respectively. The RF classifier shows slight improvement (the OA increases by 1.2%, the AA by 1.19%, and the f1 score by 0.017), while the XGBoost classifier shows the least improvement (the OA increases by 0.84%, the AA by 0.89%, and the f1 score by 0.0127). Notably, the combination of all the features (P + SS + CCS + P2) shows the best performance for three classifiers: the PLR, SVM, and RF. For the XGBoost classifier, (P + P2) and (P + SS + CCS + P2) show similar performance. Interestingly, (P + SS) or (P + SS + CCS) show similar performance for all four (PLR, SVM, RF, and XGBoost) classifiers.

When the easy-negative and hard-negative extended datasets are mixed and tested together, the addition of the P2 features on top of the P features (P + P2) and the combination of all the features (P + SS + CCS + P2) improve the predictive performance for all four classifiers over all three metrics, the OA, AA and f1 score, as shown in Table 5. Overall, (P + P2) and (P + SS + CCS + P2) show the best performance across all the classifiers. When all the features are combined (P + SS + CCS + P2), the OA is increased by 1.64%, the AA by 1.56%, and the f1 score by 0.0421 for the PLR classifier. For the SVM classifier, the increases are 1% for the OA, 0.72% for the AA, and 0.0245 for the f1 score. Similar increases are obtained for the RF and XGBoost classifiers, with an increase in the OA by 0.62%, the AA by 1.14%, and the f1 score by 0.0211, and the OA by 0.72%, the AA by 1.05%, and the f1 score by 0.0218, respectively.

### 2.4. A Comparison of Our Model Performance to Previously Published Models

Overall, the RF classifier has the best performance in predicting conotoxins from non-toxic or other toxic peptides across multiple datasets. Table 6 shows how our model performance compares to previously published models (i.e., TOXIFY [9], ToxClassifier [10], ClanTox [11], ToxinPred [12], PredCSF [13]). When the primary sequence is used as the only feature, our model outperforms the best-performing published model, ToxinPred, by 1.74% in OA and 0.1% in Recall. When adding P2 on top of the primary amino acid sequence feature, our model outperforms ToxinPred by 2.27% in OA and 0.78% in recall.

## 3. Discussion

We have demonstrated that, contrary to the current practice of using only the primary sequence (P) feature, the inclusion of PTM information as well as CCS values, when coupled with additional structural features, improves the prediction accuracy of conotoxins against non-toxic and other toxic peptides across varied datasets and across four different commonly used ML classifiers (PLR, SVM, RF, and XGBoost). In particular, the addition of these new features improved the PLR classifier significantly, with an overall accuracy increase of ~93% to ~97%, while the average accuracy increased from ~90% to 95%, and the f1 score increased from 0.8603 to 0.9271 when predicting conotoxins from non-toxic samples (the extended easy-negative dataset). The fact that all four classifiers converge to similar final accuracies and f1 scores indicates that the addition of new features increases both prediction accuracy and confidence when predicting conotoxins from non-toxic peptides. Furthermore, the performance of the RF and XGBoost classifiers is slightly better than the other two classifiers (PLR and SVM, which have similar performance) across different datasets, suggesting that either the RF or XGBoost classifier can be used successfully to build the final model for conotoxin prediction. However, the RF classifier seems to be a better choice due to its consistently higher performance across various datasets.

Our findings also suggest that there are conserved chemical and structural signatures across conotoxins that distinguish them from non-toxic peptides and other kinds of toxins. The acquisition of new, additional experimental data on isomer conformations of conotoxins would be helpful to expand the training datasets and to bolster the impact of CCS and structural features. Our results also imply the existence of similar chemical and structural signatures in other toxin families and that an ML platform that predicts different kinds of toxins and their toxicity is feasible. Additionally, traditional structure–function relationships suggest that such features can also be used for the prediction of receptor binding partners.

## 4. Materials and Methods

### 4.1. Construction of Datasets

Conotoxin data were extracted from the Conoserver [33], Protein Data Bank [34], and Biological Magnetic Resonance Bank [35] websites. Both easy- and hard-negative datasets were constructed using peptide samples with lengths equal to or less than 80 residues and with known three-dimensional structures. The easy-negative dataset includes samples that are not toxic, while the hard-negative dataset consists of toxic peptides, including spider, scorpion, and snake toxins. Entries containing post-translational modifications were included in all the datasets.

Negative datasets were obtained from the Protein Data Bank using keyword searches. The easy-negative dataset was built using the search term “NOT toxic”, and the hard-negative dataset was constructed using the search terms “toxic” and “species NOT conus”, and entries were limited to peptides with a maximum length of 80 amino acids. Entries with the keywords “Synthetic”, “Unknown function”, or “De Novo” were all removed from the datasets. Entries that were conantokins, prions, antifungal, or antimicrobial were placed in the hard-negative dataset regardless of other identifying tags.

### 4.2. Feature Extraction

**Figure 3:**
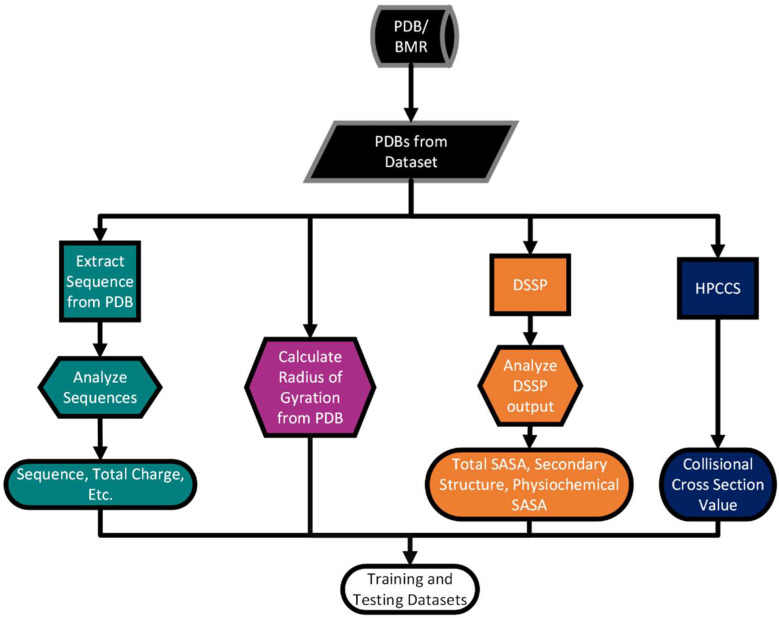
Workflow for feature extraction.

The general workflow for feature extraction from PDB/structure files is shown in Figure 3. Basically, the amino acid sequence of an entry is extracted from the PDB/structure file, and the corresponding structural features are then extracted from the PDB/structure file using the DSSP [28] program.

The thirteen features extracted from the datasets, belonging to three general categories, were divided into four groups (P, P2, SS, and CCS), as shown in Figure 2. Within the compositional category, primary sequence features include amino acid composition, such as the frequency of occurrence, and the g-gap dipeptide feature to reflect the position of each amino acid in the sequences. G-gap peptide features count the frequencies of co-occurring residues in the sequence as neighbors with a g-residue separating them. We used g = 0, 1, 2 to extract amino acid positional information with adjacent amino acids or with one or two residues separating them, respectively. Physiochemical features include the charge, mass, relative size, and relative polarity (aliphatic, aromatic, polar, hydrophobic, positively charged, negatively charged) of each residue, as previously described [24]. For non-standard amino acids, the physiochemical features were manually assigned based on their modifications, i.e., non-polar sidechains modified by the addition of an alcohol were reassigned as polar, as with hydroxyproline. A similar analysis, as conducted for the standard amino acids, was performed.

Structural features include secondary structure information, the area exposed to solvent within each physiochemical class, the radius of gyration, and CCS values. The DSSP software (version 3.0.0) [27,28,36,37] was used to calculate the secondary structure type and solvent-eposed surface area for each amino acid. The software package HPCCS (version 1.0) [22] was used to calculate CCS values based on the masses and partial charges determined by pdb2pqr (version 2.1.1) [37,38] for each atom in every input PDB file. For PTMs, custom parameters were employed based on the amber force field [39]. The Cα positions were used to calculate the radius of gyration.

### 4.3. Feature Selection Procedure

#### 4.3.1. F-Score

The goal of the feature selection step is to find the best feature subsets to maximize the robustness and the performance of the classifiers, and this will be discussed below in more detailed. Here we use F-score, a variance-based analysis, which measures the classifying power of the features, where the larger the F-score, the higher its classifying power. For each feature, the F-score is calculated as follows:

$$F\left(feature\right)=\frac{variance\;between\;classes}{variance\;within\;classes}$$

#### 4.3.2. Elimination of Highly Correlated Features

Before applying the feature selection protocol to the data, highly correlated features were removed from the dataset. If this was not carried out, the feature selection method would fail and select only a group of highly correlated features, which would negatively affect the classifier’s performance. To address this issue, Pearson correlation coefficients are computed between features to measure redundancy. If the correlation coefficient between two features is larger than a preset threshold, the one with the smaller F-score is removed. The preset threshold is a hyperparameter, whose value is highly dependent on the datasets. The output of this process produces a smaller, but more independent, set of features, which improves the classifier’s performance.

#### 4.3.3. Incremental Feature Selection

The incremental feature selection framework [40] was employed to select the optimal feature subset used to build the classifiers. All features were ranked using the F-score as described above (Section 4.3.1), and redundant features were removed. All subsets are formed from a range of features, and an ML method is used to measure their performance with cross-validation. The simple linear SVM is used since the dataset size is small. The optimum number of features is the one that produces the highest balanced accuracy. Since our datasets are imbalanced, with more entries in the negative dataset compared to the positive dataset, the balanced accuracy, which is the average accuracy weighted by the classes’ size, is a better metric to evaluate the classifier’s performance than the overall accuracy.

### 4.4. Classifiers

**Figure 4:**
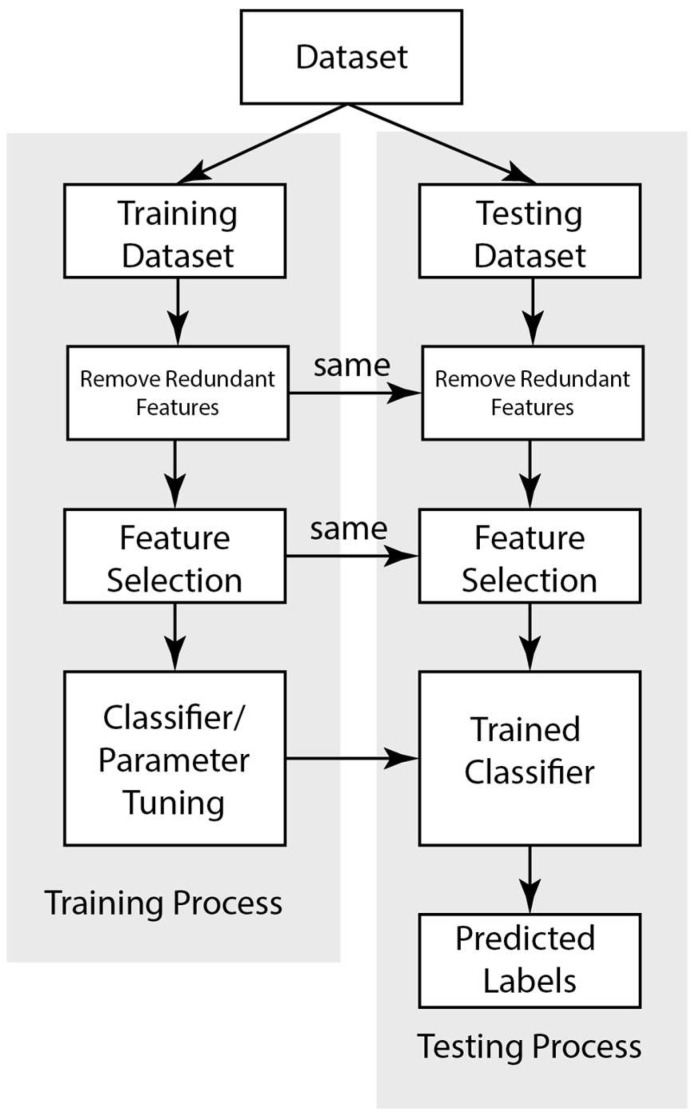
Overall ML pipeline describing the process of using a dataset to train and cross-validate a classifier.

In this study, four common ML classifiers: PLR [29], SVM [30], RF [31], and XGBoost [32], were employed to evaluate how new features would affect the prediction accuracy for the conotoxins. Given that our datasets are small, the aim was to keep our models at low complexity to make the models more robust [41]. A linear kernel was used for the SVM. As shown in Figure 4, the classifiers were trained using the training data, meaning their parameters are optimized for the classifiers to fit the training data. The trained classifier’s performance was then tested on the testing dataset. The trained classifier, with its optimized parameters, was then used to make predictions on data with unknown labels.

### 4.5. Using Geometric SMOTE to Handle Imbalanced Datasets

When classifying conotoxins against the mixed extended negative datasets (easy-negative + hard-negative or non-toxic + other toxic peptides), the data were highly imbalanced because the sample size of the negative dataset (877 entries) was much larger than the sample size of known conotoxins (184 entries). Besides using the average accuracy metrics, for better evaluation, an over-sampling technique called Geometric Synthetic Minority Oversampling Technique (GSMOTE) [42] was used to correct for this issue. Oversampling techniques are general approaches that address the imbalanced datasets by generating artificial data for the minority class. The SMOTE [43] class of methods generates synthetic samples along the line segments that connect minority class samples, which fills in the gap between minority class samples and densifies the minority clusters. GSMOTE is a variant of SMOTE that generates synthetic data, specifically in *a geometric region* of the minority samples. In this way, *noisy* samples from the minority class are added to the data, which increases the sample size without duplicating the samples in the classes.

### 4.6. Performance Evaluation

To maintain consistency across multiple datasets, a leave-one-out cross-validation protocol was used to measure the performance of the classifiers. The metrics used to measure the classification performance were the overall accuracy (OA), average accuracy (AA), precision (Pr), recall (Re), and f1 score [44], which are defined as follows:

$$OA=\frac{TP_{0}+TP_{1}}{N}\;\;\;where\;N-total\;number\;of\;samples$$

$$AA=\frac{\left(Sn_{0}+Sn_{1}\right)}{2}\;$$

$$Pr=\frac{{TP}_{i}}{{TP}_{i}+{FP}_{i}}\;\;\;i=0,1$$

$$Re=\frac{TP_{i}}{TP_{i}+FN_{i}}\;\;\;i=0,1$$

$$f1=\frac{2\times Pr\times Re}{Pr+Re}$$

where $TP_{i}$ and $FN_{i}$ are the true positives and false negatives for the $i^{th}$ class.

### 4.7. Machine Learning Pipeline

An overall ML pipeline is shown in Figure 4 and illustrates how the dataset was used to train and cross-validate a classifier. The three main steps in our pipeline (feature selection, classifier training, and prediction) were used on the testing data as indicated. For consistency, only the leave-one-out cross-validation (LOOCV) protocol was implemented throughout all the classifying tasks. The feature selection and redundant feature removal steps were implemented on each of the training datasets. For imbalanced datasets, the oversampling step was also applied to the training set.

The optimal number of features was determined using the incremental feature selection framework. Each classifier also had some hyperparameters that needed to be tuned. For the SVM and PLR, only the regularization parameters were tuned, and these parameters were automatically determined through the cross-validation within the training process. The trained classifier was then applied and tested against samples to predict their label as a conotoxin or not. The overall performance was evaluated using the OA, AA, and f1 score metrics.

## Figures and Tables

**Table 1:** Number of samples in small and extended datasets used in the machine learning experiments.

Dataset Names	Number of Samples	Dataset Names	Number of Samples
Small positive	154	Extended positive	184
Small easy-negative	180	Extended easy-negative	560
Small hard-negative	178	Extended hard-negative	317

**Table 2:** The average accuracy and f1 scores of classification performance across different datasets.

Datasets		Feature Sets							
		P		P + SS + CCS		P + P2		P + SS + CCS + P2	
		AA	f1	AA	f1	AA	f1	AA	f1
**Small Datasets**	Conotoxins vs. Easy-negative	0.9457	0.9272	0.9311	0.9236	0.9468	0.9338	0.9422	0.9302
	Conotoxins vs. Hard-negative	0.9376	0.9428	0.9437	0.9392	0.9550	0.9498	0.9489	0.9431
	Conotoxins vs. Easy + Hard-negative	0.9231	0.8917	0.9237	0.8967	0.9337	0.9045	0.9364	0.9170
**Extended Datasets**	Conotoxins vs. Easy-negative	0.9490	0.9167	0.9504	0.9080	0.9483	0.9290	0.9571	0.9271
	Conotoxins vs. Hard-negative	0.9308	0.9089	0.9292	0.9135	0.9396	0.9200	0.9387	0.9259
	Conotoxins vs. Easy + Hard-negative	0.9052	0.8824	0.9185	0.8854	0.9314	0.9027	0.9333	0.9035

**Table 3:** The classification performance of different feature sets for the extended conotoxins versus the extended easy-negative dataset (non-toxic peptides). Performance of the PLR and SVM classifiers (top table) and the RF and XGBoost classifiers (bottom table) is show.

Classifier	PLR					SVM				
Feature Sets	OA	AA	Recall	Precision	f1	OA	AA	Recall	Precision	f1
**P**	0.9328	0.8978	0.8280	0.8953	0.8603	0.9355	0.9134	0.8735	0.8430	0.8580
**SS**	0.8884	0.8344	0.6996	0.9070	0.7899	0.8884	0.8344	0.6978	0.9128	0.7909
**SS + CCS**	0.8925	0.8392	0.7054	0.9186	0.7980	0.8804	0.8252	0.6797	0.9128	0.7792
**P + CCS**	0.9368	0.9058	0.8453	0.8895	0.8669	0.9341	0.9021	0.8398	0.8837	0.8612
**P + SS**	0.9462	0.9230	0.8793	0.8895	0.8844	0.9530	0.9303	0.8870	0.9128	0.8997
**P + SS + CCS**	0.9570	0.9365	0.8977	0.9186	0.9080	0.9476	0.9216	0.8715	0.9070	0.8889
**P + P2**	0.9543	0.9389	0.9107	0.8895	0.9000	0.9570	0.9444	0.9217	0.8895	0.9053
**SS + P2**	0.9328	0.8931	0.8081	0.9302	0.8649	0.9368	0.8993	0.8205	0.9302	0.8719
**CCS + SS + P2**	0.9261	0.8836	0.7910	0.9244	0.8525	0.9328	0.8924	0.8050	0.9360	0.8656
**P + SS + CCS + P2**	0.9664	0.9536	0.9298	0.9244	0.9271	0.9610	0.9444	0.9133	0.9186	0.9159
**Classifier**	**RF**					**XGBoost**				
**Feature Sets**	**OA**	**AA**	**Recall**	**Precision**	**f1**	**OA**	**AA**	**Recall**	**Precision**	**f1**
**P**	0.9624	0.9540	0.9390	0.8953	0.9167	0.9489	0.9312	0.8988	0.8779	0.8882
**SS**	0.9355	0.9080	0.8563	0.8663	0.8613	0.9382	0.9130	0.8663	0.8663	0.8663
**SS + CCS**	0.9435	0.9206	0.8779	0.8779	0.8779	0.9328	0.9030	0.8466	0.8663	0.8563
**P + CCS**	0.9597	0.9501	0.9329	0.8895	0.9107	0.9462	0.9273	0.8929	0.8721	0.8824
**P + SS**	0.9610	0.9511	0.9333	0.8953	0.9139	0.9597	0.9449	0.9176	0.9070	0.9123
**P + SS + CCS**	0.9556	0.9416	0.9162	0.8895	0.9027	0.9543	0.9357	0.9012	0.9012	0.9012
**P + P2**	0.9677	0.9599	0.9458	0.9128	0.9290	0.9610	0.9493	0.9281	0.9012	0.9145
**SS + P2**	0.9570	0.9462	0.9268	0.8837	0.9048	0.9530	0.9361	0.9053	0.8895	0.8974
**CCS + SS + P2**	0.9597	0.9520	0.9383	0.8837	0.9102	0.9503	0.9308	0.8947	0.8895	0.8921
**P + SS + CCS + P2**	0.9651	0.9579	0.9451	0.9012	0.9226	0.9624	0.9522	0.9337	0.9012	0.9172

**Table 4:** The classification performance of different feature sets for the extended conotoxins versus the extended hard-negative dataset (other toxic peptides). Performance of the PLR and SVM classifiers (top table) and the RF and XGBoost classifiers (bottom table) is shown.

Classifier	PLR					SVM				
Feature Sets	OA	AA	Recall	Precision	f1	OA	AA	Recall	Precision	f1
**P**	0.9159	0.9021	0.8580	0.8978	0.8740	0.9205	0.9033	0.8488	0.9164	0.8786
**SS**	0.8737	0.8787	0.8946	0.7800	0.8301	0.8716	0.8789	0.9022	0.7727	0.8294
**SS + CCS**	0.8723	0.8779	0.8958	0.7757	0.8287	0.8694	0.8766	0.8996	0.7690	0.8262
**P + CCS**	0.9171	0.9059	0.8701	0.8908	0.8770	0.9213	0.9062	0.8582	0.9115	0.8810
**P + SS**	0.9229	0.9139	0.8856	0.8937	0.8867	0.9225	0.9126	0.8812	0.8954	0.8857
**P + SS + CCS**	0.9212	0.9118	0.8820	0.8931	0.8843	0.9227	0.9132	0.8829	0.8961	0.8864
**P + P2**	0.9290	0.9180	0.8831	0.9120	0.8943	0.9344	0.9222	0.8835	0.9260	0.9014
**SS + P2**	0.9153	0.9161	0.9189	0.8521	0.8819	0.9112	0.9130	0.9190	0.8434	0.8772
**CCS + SS + P2**	0.9162	0.9166	0.9183	0.8547	0.8831	0.9075	0.9109	0.9217	0.8353	0.8734
**P + SS + CCS + P2**	0.9314	0.9242	0.9015	0.9035	0.8995	0.9356	0.9264	0.8974	0.9176	0.9048
**Classifier**	**RF**					**XGBoost**				
**Feature Sets**	**OA**	**AA**	**Recall**	**Precision**	**f1**	**OA**	**AA**	**Recall**	**Precision**	**f1**
**P**	0.9386	0.9289	0.8979	0.9237	0.9089	0.9347	0.9256	0.8968	0.9152	0.9031
**SS**	0.9165	0.9091	0.8859	0.8786	0.8793	0.9129	0.9033	0.8727	0.8790	0.8728
**SS + CCS**	0.9120	0.9052	0.8835	0.8696	0.8733	0.9125	0.9038	0.8766	0.8754	0.8727
**P + CCS**	0.9399	0.9305	0.9005	0.9261	0.9103	0.9358	0.9258	0.8941	0.9212	0.9049
**P + SS**	0.9395	0.9289	0.8951	0.9297	0.9096	0.9339	0.9243	0.8938	0.9158	0.9019
**P + SS + CCS**	0.9424	0.9315	0.8970	0.9350	0.9135	0.9340	0.9247	0.8954	0.9151	0.9025
**P + P2**	0.9463	0.9364	0.9051	0.9396	0.9199	0.9440	0.9367	0.9134	0.9265	0.9175
**SS + P2**	0.9428	0.9344	0.9074	0.9275	0.9154	0.9222	0.9147	0.8908	0.8892	0.8867
**CCS + SS + P2**	0.9392	0.9314	0.9070	0.9187	0.9104	0.9229	0.9153	0.8910	0.8899	0.8877
**P + SS + CCS + P2**	0.9506	0.9408	0.9092	0.9467	0.9259	0.9431	0.9345	0.9070	0.9289	0.9158

**Table 5:** The classification performance of different feature sets for the extended conotoxins versus the mixed extended negative dataset (easy-negative + hard-negative or non-toxic + other toxic peptides). Performance of the PLR and SVM classifiers (top table) and of the RF and XGBoost classifiers (bottom table) is shown.

Classifier	PLR					SVM				
Feature Sets	OA	AA	Recall	Precision	f1	OA	AA	Recall	Precision	f1
**P**	0.9370	0.9159	0.8848	0.7700	0.8197	0.9403	0.9190	0.8877	0.7805	0.8273
**SS**	0.8840	0.8916	0.9028	0.5980	0.7166	0.8737	0.8850	0.9017	0.5722	0.6979
**SS + CCS**	0.8814	0.8899	0.9024	0.5908	0.7113	0.8759	0.8882	0.9063	0.5777	0.7032
**P + CCS**	0.9436	0.9199	0.8851	0.7963	0.8352	0.9453	0.9228	0.8896	0.8020	0.8402
**P + SS**	0.9469	0.9253	0.8934	0.8054	0.8444	0.9467	0.9248	0.8925	0.8048	0.8433
**P + SS + CCS**	0.9437	0.9260	0.8998	0.7901	0.8380	0.9458	0.9292	0.9047	0.7965	0.8443
**P + P2**	0.9535	0.9276	0.8894	0.8371	0.8599	0.9524	0.9234	0.8808	0.8376	0.8554
**SS + P2**	0.9191	0.9162	0.9120	0.6919	0.7841	0.9163	0.9195	0.9242	0.6818	0.7821
**CCS + SS + P2**	0.9201	0.9203	0.9205	0.6949	0.7891	0.9105	0.9182	0.9295	0.6611	0.7705
**P + SS + CCS + P2**	0.9534	0.9315	0.8992	0.8322	0.8618	0.9503	0.9262	0.8907	0.8242	0.8518
**Classifier**	**RF**					**XGBoost**				
**Feature Sets**	**OA**	**AA**	**Recall**	**Precision**	**f1**	**OA**	**AA**	**Recall**	**Precision**	**f1**
**P**	0.9658	0.9087	0.8248	0.9579	0.8824	0.9611	0.9161	0.8499	0.9078	0.8743
**SS**	0.9516	0.8771	0.7676	0.9209	0.8324	0.9466	0.8881	0.8020	0.8631	0.8267
**SS + CCS**	0.9505	0.8718	0.7560	0.9222	0.8274	0.9431	0.8798	0.7866	0.8538	0.8141
**P + CCS**	0.9670	0.9092	0.8242	0.9667	0.8876	0.9642	0.9214	0.8584	0.9167	0.8836
**P + SS**	0.9666	0.9064	0.8179	0.9711	0.8844	0.9646	0.9223	0.8600	0.9188	0.8850
**P + SS + CCS**	0.9659	0.9052	0.8158	0.9665	0.8819	0.9646	0.9224	0.8604	0.9186	0.8854
**P + P2**	0.9716	0.9188	0.8409	0.9811	0.9027	0.9702	0.9336	0.8798	0.9342	0.9033
**SS + P2**	0.9673	0.9139	0.8352	0.9585	0.8895	0.9583	0.9051	0.8269	0.9089	0.8621
**CCS + SS + P2**	0.9664	0.9103	0.8277	0.9588	0.8849	0.9610	0.9137	0.8441	0.9108	0.8730
**P + SS + CCS + P2**	0.9720	0.9201	0.8438	0.9789	0.9035	0.9683	0.9266	0.8652	0.9363	0.8961

**Table 6:** A comparison of our model performance to previously published models.

Method	Training Set	Acc	Recall	Types of Features	Usage
This paper (RF)	See Methods	0.9624	0.9390	Sequence	Used to predict conotoxins from non-toxic peptides
This paper (RF)	See Methods	0.9677	0.9458	Sequence and PTMs	Used to predict conotoxins from non-toxic peptides
TOXIFY [9]	Swiss-Prot-derived	0.8600	0.7600	Sequence	Used to predict if a peptide is toxic
ToxClassifier [10]	Swiss-Prot-derived	0.7700	0.5600	Sequence	Used to predict if a peptide is toxic
ClanTox [11]	Swiss-Prot-derived	0.6800	0.5400	Sequence	Used to predict if a peptide is toxic
ToxinPred [12]	Composite dataset	0.9450	0.9380	Sequence	Used to predict if a peptide is toxic
Pred-CSF [13]	S2	0.9065	0.8793	Sequence	Used to predict conotoxin super families

Comparative table of prediction results from multiple models showing the accuracy and recall of different methods. Training sets used by the above methods include an S2 dataset, a Swiss-Prot-derived dataset, and a composite dataset. The S2 dataset is a superfamily training set containing 261 entries with four superfamilies: A (63 samples), M (48 samples), O (95 samples), and T (55 samples). The Swiss-Prot-derived dataset consists of Swiss-Prot entries, as described in [9]. The composite dataset consists of small toxins from several different databases, with entries having more than 35 residues and any non-natural amino acids removed.

## Data Availability

Six datasets used in this study along with all the features extracted are available in File S1.

## Supplementary Materials

The following supporting information can be downloaded at https://www.mdpi.com/article/10.3390/toxins15110641/s1: Figure S1: Distribution of conotoxin classes; Figure S2: Solvent exposure; Table S1: HPCCS parameters for common post-translational modifications found in conotoxins; Table S2: List of physiochemical classes (left) and amino acids within each class (right); Table S3: DSSP secondary structure definitions; Table S4: Feature sets; File S1: Six datasets used in this study along with all features extracted.
