# Supplementary material for: Conotoxin Prediction: New Features to Increase Prediction Accuracy
Source: Toxins (Basel). 2023 Nov 3;15(11):641. doi: 10.3390/toxins15110641 (PMC10675404; doi:10.3390/toxins15110641)
Supplement: Supplementary file 1 [file toxins-15-00641-s001.zip › toxins-2632921-supplementary.pdf]

Supplementary Materials: Conotoxin Prediction: New Features to Increase Prediction Accuracy

Lyman K. Monroe, Duc P. Truong, Jacob C. Miner, Samantha H. Adikari, Zachary J. Sasiene, Paul W. Fenimore, Boian Alexandrov, Robert F. Williams, and Hau B. Nguyen

Table S1. HPCCS parameters for common post-translational modifications found in conotoxins.

Post-Translational Modification	HPCCS parameters
Hydroxyproline	PCA N -0.465 1.550 N
	PCA CA -0.065 1.700 CT
	PCA CB -0.324 1.700 CT
	PCA CG -0.454 1.700 CT
	PCA CD 0.827 1.700 C
	PCA OE -0.766 1.520 O
	PCA C 0.518 1.700 C
	PCA O -0.716 1.520 O
	PCA HA 0.263 1.200 H1
	PCA HB2 0.302 1.200 HC
	PCA HB3 0.265 1.200 HC
	PCA HG2 0.314 1.200 HC
	PCA HG3 0.301 1.200 HC
Pyroglutamic acid	HYP N -0.404 1.550 N
	HYP CA -0.061 1.700 CT
	HYP C 0.472 1.700 C
	HYP O -0.741 1.520 O
	HYP CB -0.352 1.700 CT
	HYP CG 0.142 1.700 CT
	HYP CD -0.226 1.700 CT
	HYP OD1 -0.917 1.520 OH
	HYP HA 0.250 1.200 H1
	HYP HB2 0.286 1.200 HC
	HYP HB3 0.293 1.200 HC
	HYP HG 0.226 1.200 H1
	HYP HD22 0.283 1.200 H1
	HYP HD23 0.267 1.200 H1
	HYP HD1 0.482 1.200 HO
Carboxyglutamic acid	CGU N -0.297 1.550 N
	CGU CA -0.013 1.700 CT
	CGU C 0.649 1.700 CT
	CGU O -0.652 1.520 O
	CGU CB -0.261 1.700 CT
	CGU CG -0.413 1.700 CT
	CGU CD1 1.174 1.700 C
	CGU CD2 1.171 1.700 C
	CGU OE11 -0.673 1.520 O2
	CGU OE12 -0.780 1.520 O2

	CGU OE21 -0.695 1.520 O2<br>CGU OE22 -0.764 1.520 O2<br>CGU HA 0.376 1.200 H1<br>CGU HB2 0.411 1.200 HC<br>CGU HB3 0.369 1.200 HC<br>CGU HG 0.398 1.200 HC
-NH2	NH2 N -0.463 1.824 N<br>NH2 HN1 0.231 0.600 H<br>NH2 HN2 0.231 0.600 H

Physiochemical definitions

Table S2. List of physiochemical classes (left) and amino acids within each class (right). .
h = Hydroxyproline, p = Pyroglutamic acid, c = Carboxyglutamic acid

Physiochemical Class	Amino Acids
Charged	DKEHR
Aliphatic	ILV
Aromatic	FHWY
Polar	DERKQN
Hydrophobic	CVLIMFW
Positively Charged	KRH
Negatively Charged	DE
Tiny	GACDST
Small	EHILKMNPQV
Large	FRWY
PRM	hpc

Secondary Structure

Table S3. DSSP secondary structure definitions.

DSSP name	Definition
G	3-turn helix (310 helix). Min length 3 residues.
H	4-turn helix (alpha helix). Min length 4 residues.
I	5-turn helix (pi helix). Min length 5 residues.
T	hydrogen bonded turn (3, 4 or 5 turn)
E	beta sheet in parallel and/or anti-parallel sheet conformation (extended strand). Min length 2 residues.
B	residue in isolated beta-bridge (single pair beta-sheet hydrogen bond formation)
S	bend (the only non-hydrogen-bond based assignment)

DSSP was also used to determine the solvent accessible surface area (SASA) of each residue. The SASA of each residue was added to the total for each respective residue class as defined in table S1.

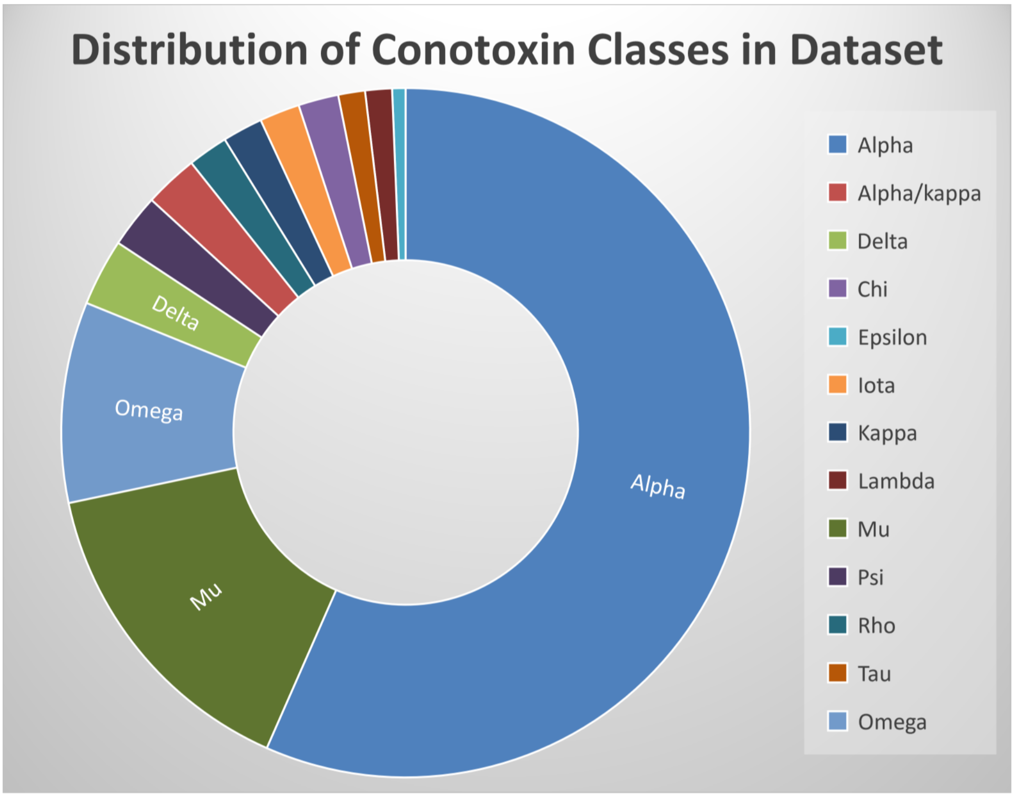

Figure S1. Distribution of conotoxin classes
Fraction of each conotoxin class in the positive dataset.

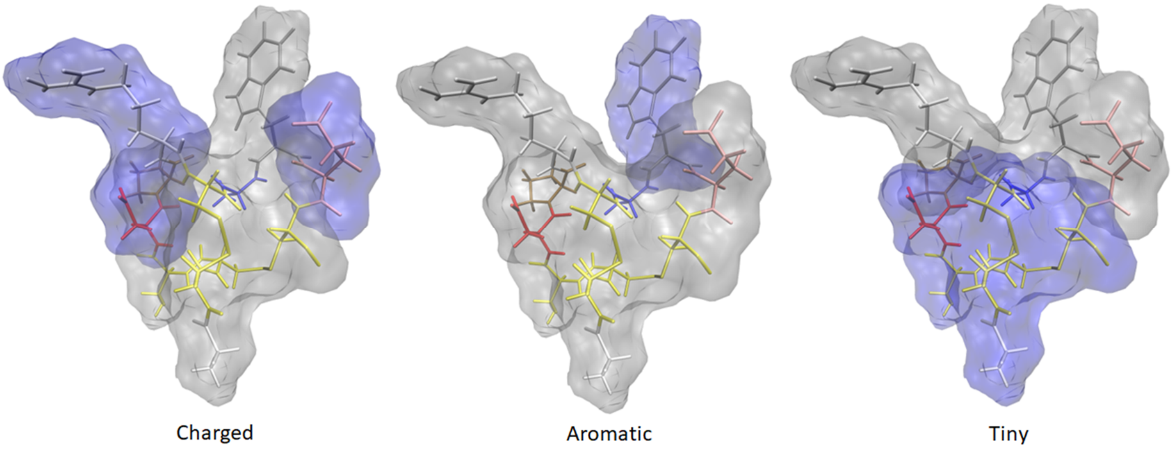

Figure S2. Solvent exposure
The conotoxin IM1 with a native disulfide binding pattern shown in a liquorish and transparent surface representation. The surface is colored in blue for charge, aromatic, and tiny residues in the left, middle and right panels respectively. Other types of surfaces are shown in silver in each panel.

Table S4. Feature sets.

Feature Set	Included features
P	Number of Charged residues.<br>Number of Aliphatic residues.<br>Number of Aromatic residues.<br>Number of Polar residues.<br>Number of Hydrophobic residues.<br>Number of Positively charged residues.<br>Number of Negatively charged residues.<br>Number of Tiny residues.<br>Number of Small residues.<br>Number of Large residues.<br>Total Charge<br>MASS(KDA)<br>Normalized amino acid counts (number of an Amino acid / sequence length)<br>Dipeptide0<br>Dipeptide1
P2	Dipeptide2<br>Number of posttranslational modifications
SS	Number of residues in the DSSP defined structures: G, H, I, T, E, B, S.<br><br>SASA of:<br><br>-Charged residues.<br>-Aliphatic residues.<br>-Aromatic residues.<br>-Polar residues.<br>-Hydrophobic residues.<br>-Positively charged residues.<br>-Negatively charged residues.<br>-Tiny residues.<br>-Small residues.<br>-Large residues.<br><br>- Posttranslational Modifications<br><br>Total SASA.<br><br>Radius of Gyration.<br><br>Number of disulfide bonds.
CCS	Calculated Collisional Cross Section.

Libraries used in this work:
Perl libraries used for feature extraction: Math
Python libraries used: sys, os, glob, numpy, itertools, pandas, collections, statistics, pickle, csv.
